# 875. Development of a Cheap, Portable Assay to Rapidly Profile Resistant Bacterial Infections

**DOI:** 10.1093/ofid/ofad500.920

**Published:** 2023-11-27

**Authors:** David Roach, Roby P Bhattacharyya

**Affiliations:** Brigham and Women's Hospital, Broad Institute of MIT and Harvard, Boston, Massachusetts; Massachusetts General Hospital, Cambridge, Massachusetts

## Abstract

**Background:**

Antimicrobial resistant (AMR) bacterial infections pose a significant and growing health threat worldwide. The burden of these resistant infections is often most severe in lower income areas, where the medical infrastructure necessary for their diagnosis is lacking. The development of cost-effective and accessible diagnostic tools for the rapid identification of resistant infections in low-resource settings is crucial. Specific High-sensitivity Enzymatic Reporter Unlocking, or SHERLOCK, is a promising CRISPR-based system that can aid in the diagnosis of infections and support clinical decision making. Here, we present an assay termed BADLOCK (Bacteria and AMR Detection by SHERLOCK) that uses SHERLOCK technology to rapidly identify bacterial species and AMR genes on blood cultures.

SHERLOCK Schematic

**Figure:**
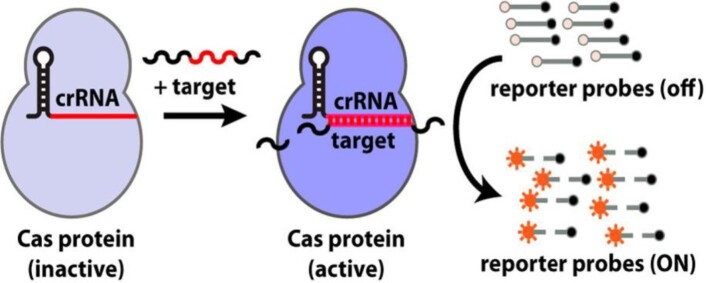

Enzymatic activity of the Cas protein is turned on when the target gene is bound, which then activates reporter probes.

**Methods:**

We combine isothermal amplification of gene targets with SHERLOCK-based detection into a single step, allowing for a streamlined workflow and a total time-to-detection of less than two hours. The assay targets the *topA* gene for species identification, which is highly conserved within species but has sufficient variability between them to allow for discrimination between even closely related pathogens. A panel of clinically important AMR genes, including CTX-M-15, *mecA*, and five canonical carbapenemases, is also targeted to provide information on resistance profiles.

BADLOCK Targets

**Figure:**
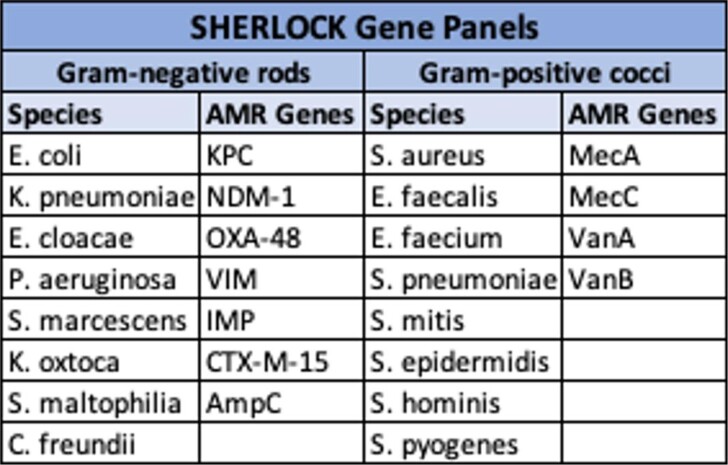

Gene targets included in the BADLOCK assay.

**Results:**

BADLOCK has high sensitivity and specificity for both AMR genes and species identification and leverages a lateral-flow platform to provide a simple visual readout. We are currently validating BADLOCK on a library of several hundred clinical isolates with known genetic backgrounds, including a large collection of carbapenem-resistant *Enterobacterales,* which are major contributors to the global burden of resistant infections.

Lateral Flow Assay Results

**Figure:**
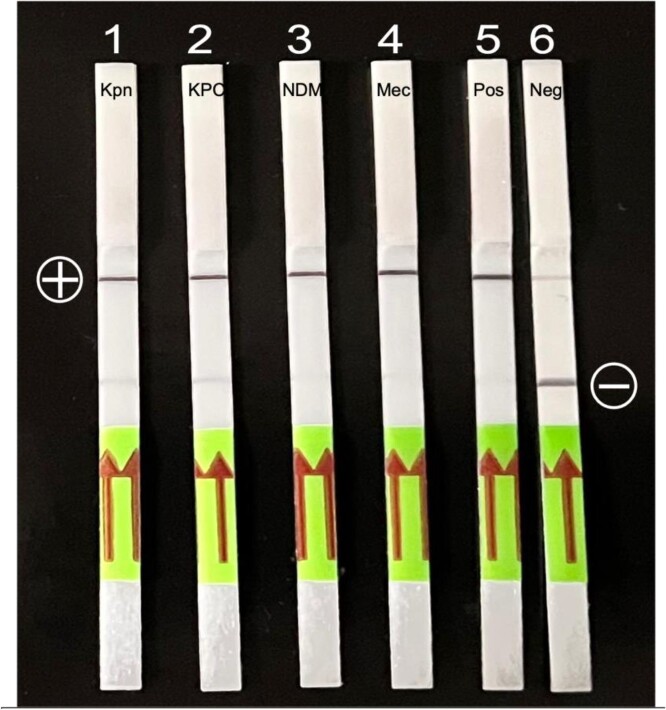

Lateral flow results. Strip 1 is the topA gene specific to K. pneumoniae. Strips 2-4 are AMR genes KPC, CTX-M-15, and MecA. Lane 5 is a positive control; lane 6 is a NTC with bottom banding. “+” and “-” symbols mark representative readouts.

**Conclusion:**

We have developed an assay for the cheap, rapid detection of bacterial infections of the bloodstream capable of detecting common pathogens as well as a panel of AMR genes. Our work has the potential to improve the diagnostic landscape for resistant infections in low-and-middle-income countries, which would represent a major step forward in infectious disease treatment in these underserved areas.

**Disclosures:**

**All Authors**: No reported disclosures

